# Formal Assignation of the Kissing Bug *Triatoma lecticularia* (Hemiptera: Reduviidae: Triatominae) to the Genus *Paratriatoma* †

**DOI:** 10.3390/insects12060538

**Published:** 2021-06-10

**Authors:** Vinicius Fernandes de Paiva, Jader de Oliveira, Cleber Galvão, Silvia Andrade Justi, José Manuel Ayala Landa, João Aristeu da Rosa

**Affiliations:** 1 Departamento de Biologia Animal, Instituto de Biologia, Universidade Estadual de Campinas, Campinas 13083-862, SP, Brazil; nisso_paiva@hotmail.com; 2 Laboratório de Entomologia em Saúde Pública, Departamento de Epidemiologia, Faculdade de Saúde Pública, Universidade de São Paulo, São Paulo 01246-904, SP, Brazil; jdr.oliveira@hotmail.com; 3 Laboratório Nacional e Internacional de Referência em Taxonomia de Triatomíneos, Instituto Oswaldo Cruz, Fiocruz, Pavilhão Rocha Lima, Rio de Janeiro 21040-360, RJ, Brazil; 4 The Walter Reed Biosystematics Unit, Smithsonian Institution Museum Support Center, Suitland, MD 20746, USA; silvinhajusti@gmail.com; 5 Entomology Branch, Walter Reed Army Institute of Research, Silver Spring, MD 20910, USA; 6 Department of Entomology, Smithsonian Institution National Museum of Natural History, Washington, DC 20013, USA; 7 Independent Researcher, Pleasanton, CA 94566, USA; jmal1942@hotmail.com; 8 School of Pharmaceutical Sciences, São Paulo State University (Unesp), Araraquara 14800-903, SP, Brazil; joao.aristeu@unesp.br

**Keywords:** taxonomy, vectors, Heteroptera, Triatomini, North America

## Abstract

**Simple Summary:**

The genus *Paratriatoma* is closely related to the paraphyletic genus *Triatoma*—the most diverse and relevant in the epidemiology of Chagas disease. Molecular phylogenetic treatments consistently place the species *Triatoma lecticularia* (Stål, 1859) as a sister to the genus *Paratriatoma*. To determine its correct taxonomic assignment, we examined the morphology of several specimens, including types, and cytogenetic data for both taxa. The observations clearly support the transfer of *Triatoma lecticularia* (Stål, 1859) to the genus *Paratriatoma*, with the resulting new combination: *Paratriatoma lecticularia* (Stål, 1859) comb. nov. (Hemiptera: Heteroptera: Reduviidae: Triatominae).

**Abstract:**

The subfamily Triatominae (Hemiptera: Reduviidae) comprises hematophagous insects that are vectors of Chagas disease; including species assigned to the genera *Triatoma* and *Paratriatoma*. Initial examination of *Triatoma lecticularia* revealed the hirsuteness covering the entire body—a characteristic and striking feature of members of the genus *Paratriatoma*—and a systematic study revealed several other morphological characters that are in diagnostic alignment with *Paratriatoma*. Based on the examination of several specimens (including the lectotype), and with the additional support of molecular and cytogenetic data, we propose the formal transferal of *Triatoma lecticularia* (Stål, 1859) into the genus *Paratriatoma* with the resulting new combination: *Paratriatoma lecticularia* (Stål, 1859) comb. nov. (Hemiptera: Reduviidae: Triatominae).

## 1. Introduction

Hematophagous insects of the subfamily Triatominae can transmit Chagas disease (American Trypanosomiasis). Currently, the subfamily comprises five tribes, 18 genera and, 156 species [1,2]. The Tribe Triatomini Jeannel, 1919 includes the genera *Dipetalogaster* Usinger, 1939; *Eratyrus* Stål, 1859; *Hermanlentia* Jurberg and Galvão, 1997; *Mepraia* Mazza, Gajardo, and Jörg, 1940; *Nesotriatoma* Usinger, 1944; *Panstrongylus* Berg, 1879; *Paratriatoma* Barber, 1938; and *Triatoma* Laporte, 1832.

Of these, the paraphyletic genus *Triatoma* is the most diverse with 82 described species [1], and the most relevant in the epidemiology of Chagas disease [3]. Historically there have been disagreements concerning the subgeneric (formal and informal) rankings within *Triatoma*. Since the 1960s, species have been grouped into complexes and subcomplexes based on morphological, geographical, ecological, and, more recently by molecular data [4,5,6,7,8,9]. One of such groupings is the *Triatoma lecticularia* complex, which has been proposed to include the Neartic species—*T. lecticularia* (Stål, 1859), *T. sanguisuga* (LeConte, 1856)*, T. gerstaeckeri* (Stål, 1859), *T. indictiva* Neiva, 1912, *T. recurva* (Stål, 1868), and *T. rubida* (Uhler, 1894) [4,8]. However, some studies show that the *Triatoma lecticularia* complex is not well supported [6,10].

*Triatoma lecticularia* was originally described as *Conorhinus lecticularius,* based on specimens from Carolina (Museum Schaumburg), India Orientalis (deposited in Musei Berolinensis) [11]. *Triatoma lecticularia occulta* (Neiva, 1911) and *T. lecticularia floridana* (Usinger, 1944) were subspecific morphotypes proposed by the authors for specimens that showed a small variation in some morphological characters, such as the size of the head and eyes and the color pattern of the body [12,13]. Typical *T. lecticularia* specimens have an elongate-oval, shiny body, with the entire surface clothed by distinct decumbent hairs. Overall color piceous, with orange or orange-yellow markings on pronotum, pleura, corium, connexivum, and ventral surface of the abdomen [11]. It presents karyotype 2n = 22 (20A + XY), the same as *Paratriatoma* [14].

The genus *Paratriatoma* is closely related to *Triatoma* and can be distinguished by the ovoid shape of the head, absence of arcuate interocellar sulcus, absence of femoral spines or tubercles, and, mainly, by the remarkable hirsuteness of the body and the appendages [15]. The monotypic species, *Paratriatoma hirsuta* Barber 1938 is closely associated with pack/wood rats of the genus *Neotoma* in the Sonora Desert [16]. Five subspecies (*P. hirsuta hirsuta, P. h. kamiensis* Ryckman 1967, *P. h. papagoensis* Ryckman 1967, *P. h. pimae* Ryckman 1967, and *P. h. yumanensis* Ryckman 1967) have been described based on noted differences in coloration of the wings and head [17]. Subsequent analysis revealed that each subspecies represents a chromatic form, and is restricted to a limited geographical area [4].

In the current study, we show that the morphology of *Triatoma lecticularia* reconciled with molecular and cytogenetic data are entirely concordant with the genus *Paratriatoma* Barber, 1938 [3,18,19], and propose the new combination: *Paratriatoma lecticularia* (Stål, 1859), comb. nov. (Hemiptera: Heteroptera: Reduviidae: Triatominae).

## 2. Material and Methods

### 2.1. Material Examined

**Figure 1:**
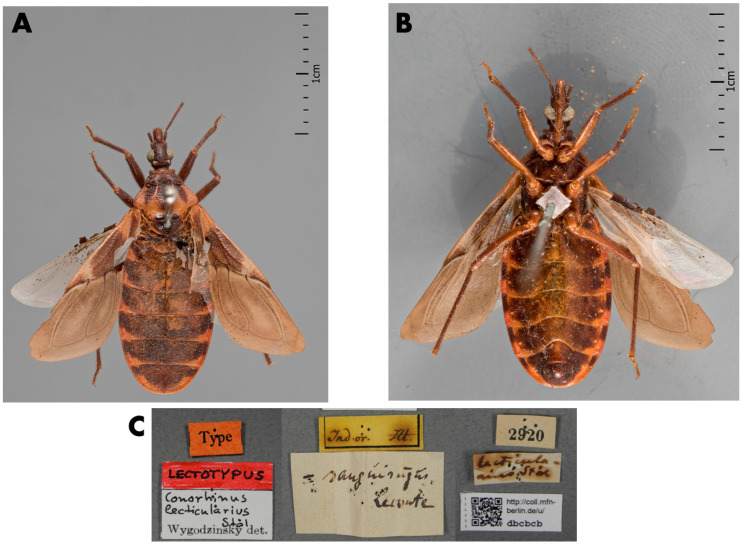
The lectotype of *Paratriatoma lecticularia* comb. nov. deposited in Natural History Museum of Berlin, Germany. (**A**) Dorsal view, (**B**) Ventral view, (**C**) Label of the specimen. At the ZMHB the photos were provided by Dr. Jürgen Deckert.

**Figure 2:**
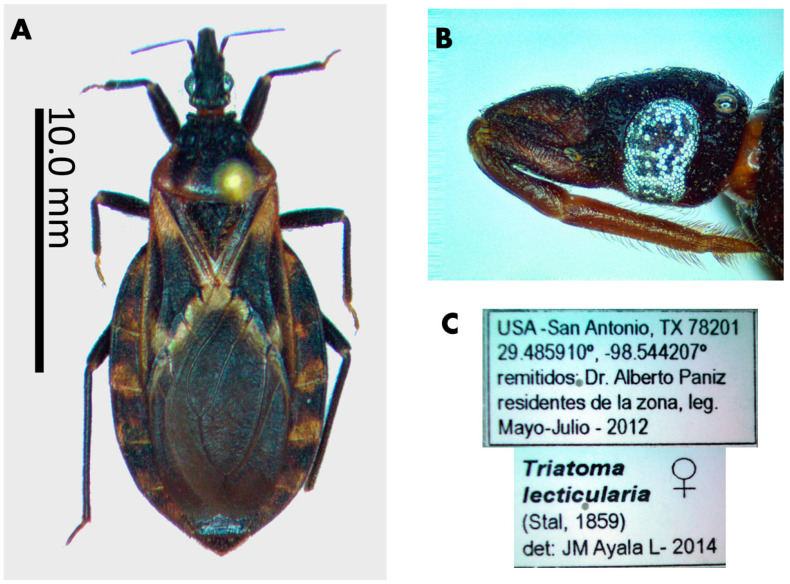
Specimen of *Paratriatoma lecticularia* from Texas, USA. (**A**) Dorsal habitus of the specimen. (**B**) Lateral view of head. (**C**) Label of the specimen.

**Figure 3:**
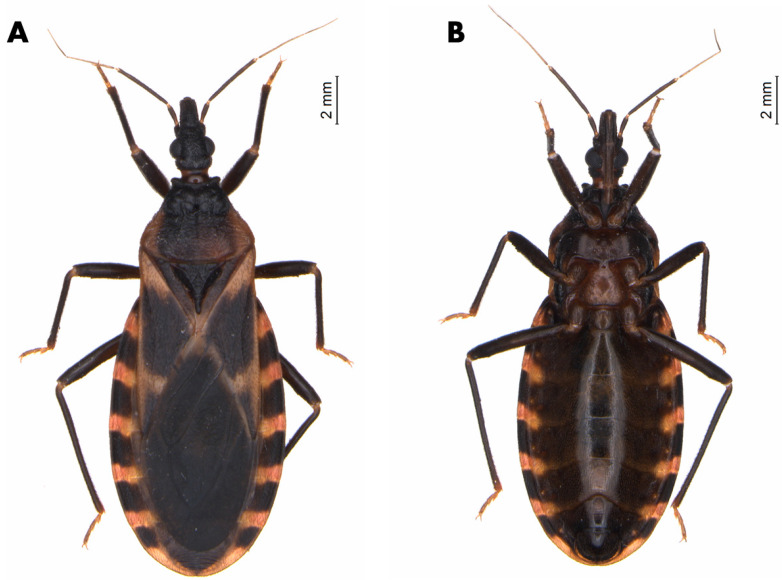
Male specimen of *Paratriatoma lecticularia* from CTJMSB CTA137. (**A**) Dorsal habitus. (**B**) Ventral view.

**Figure 4:**
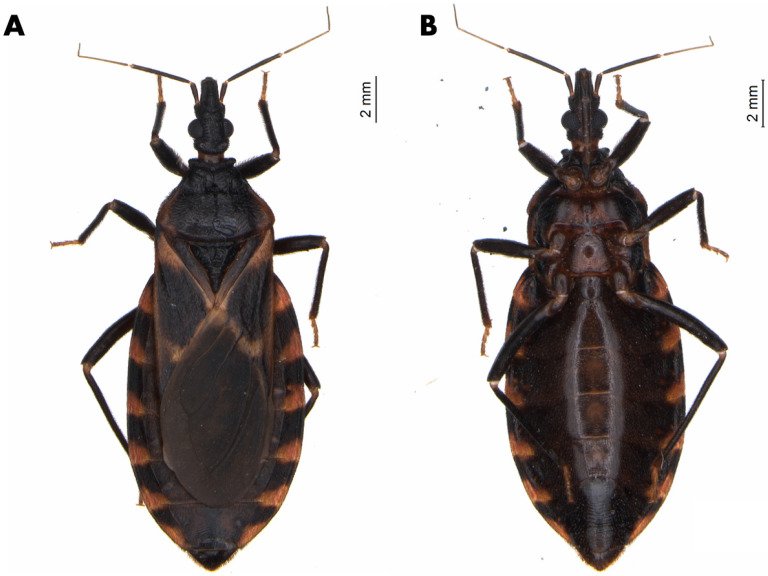
Female specimen of *Paratriatoma lecticularia* from CTJMSB CTA137. (**A**) Dorsal habitus. (**B**) Ventral view.

**Figure 5:**
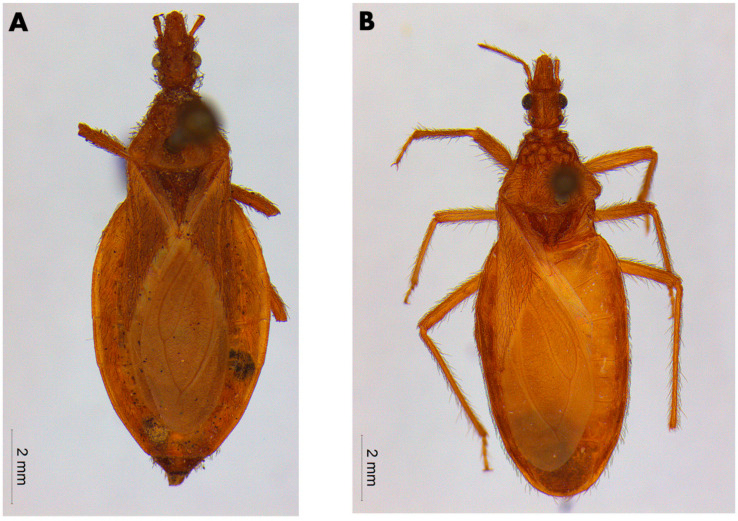
Specimens of *Paratriatoma hirsuta* from CTIOC. (**A**) Dorsal habitus of female specimen. (**B**) Dorsal habitus of male specimen.

**Figure 6:**
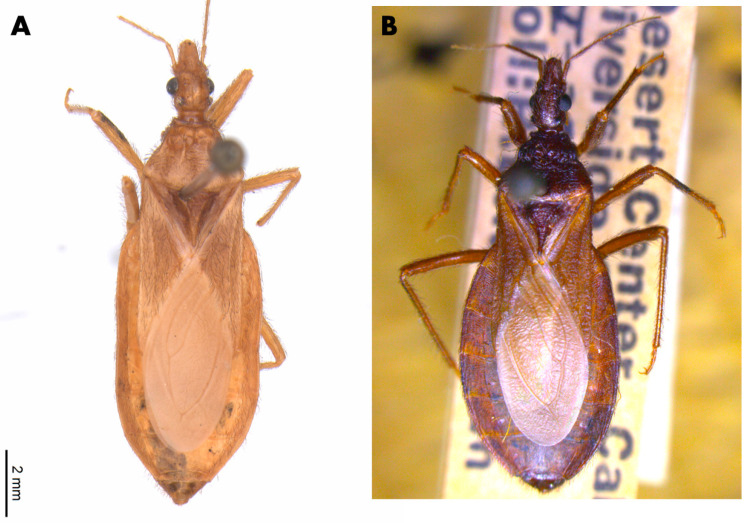
Specimens of *Paratriatoma hirsuta* from CER-USP. (**A**) Dorsal habitus of female specimen. (**B**) Dorsal habitus of a female specimen.

**Figure 7:**
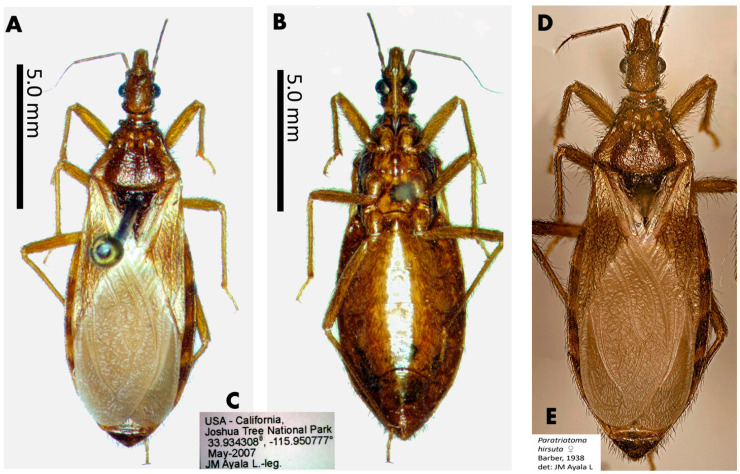
Specimens of *Paratriatoma hirsuta* from California, USA. (**A**) Dorsal habitus of the specimen. (**B**) Ventral view. (**C**) Label of the specimen. (**D**) dorsal view. (**E**) Label of the specimen.

The male lectotype of *T. lecticularia* deposited in the Humboldt Museum für Naturkunde, Berlin, Germany (Figure 1, Table 1), and five males and five females of *T. lecticularia* deposited in Triatomine collection “José Maria Soares Barata”, Universidade Estadual Paulista (UNESP), Araraquara, São Paulo, Brazil (Figure 2, Figure 3 and Figure 4, Table 1), were directly examined. The type specimen of *Paratriatoma hirsuta* deposited in U.S. National Entomological Collection, National Museum of Natural History, Smithsonian Institution, Washington DC, was also studied. Four specimens of *P. hirsuta* deposited in the “Coleção de Triatomíneos do Instituto Oswaldo Cruz, Fiocruz, Brazil” (Figure 5, Table 1), two specimens of “Coleção Entomológica de Referência, Faculdade de Saúde Pública, Universidade de São Paulo, Brazil” (Figure 6, Table 1), and ten specimens deposited in Ayala-Landa personal collection (Figure 7, Table 1), were used to compare with *T. lecticularia*.

### 2.2. Morphological Study

**Figure 8:**
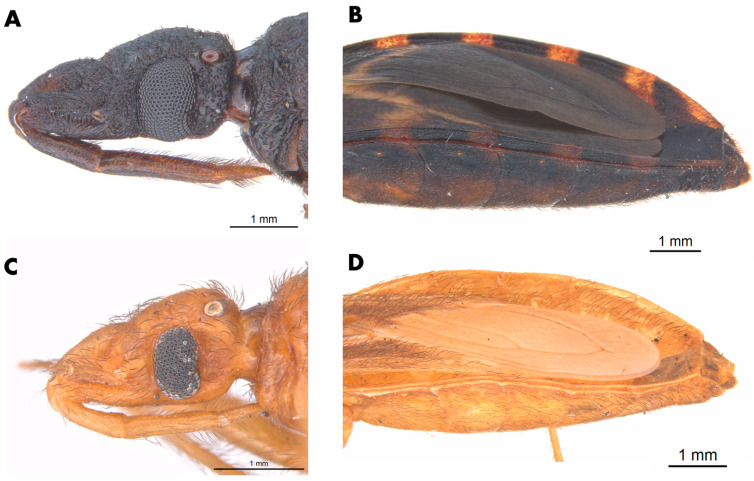
Head and conexivum of *P. lecticularia* and *P. hirsuta.* (**A**) Head in lateral view of *P. lecticularia.* (**B**) lateral view of connexivum of *P. lecticularia,* evidencing your membrane, apomorphic character of the genus. (**C**) Head in lateral view of *P. hirsuta.* (**D**) lateral view of connexivum of *P. hirsuta.*

Photographs were made using a professional camera and Leica M205C stereomicroscope and were processed with the software Leica LAS (version 4.9). Type specimens were examined through photographs, and label information is provided (Table 1). Based on Barber’s original (1938) description of the genus *Paratriatoma,* the major features distinguishing *Paratriatoma* from species in *Triatoma* include the ovoidal shape of the head, the absence of an arcuate interocellar sulcus, the absence of femoral spines or tubercles, and the intensely hirsute body and appendages. Another apomorphy is noted in the plates of the dorsal and ventral connexivum of the urosternite overlapping the mesal portion of the ventral plate of the connexivum, with a distinct membrane interpolated between the edge of the connexival plate and the lateral edge of the urosternum (Figure 8).

## 3. Results

### 3.1. Taxonomic Hierarchy

Class Insecta Linnaeus, 1758, Order Hemiptera Linnaeus, 1758, Family Reduviidae Latreille, 1807, Subfamily Triatominae Jeannel, 1919, Tribe Triatomini Jeannel, 1919, *Genus Paratriatoma lecticularia* (Stål, 1859), comb. nov. (Figure 1).

### Synonyms

*Conorhinus lecticularius* Stål, 1859 [original description]; *Conorhinus lenticularius* Stål, 1868; *Conorhinus variegatus* Stål, 1872; *Conorhinus heidemanni* Patton and Cragg, 1913; *Conorhinus occulata* Patton and Cragg, 1913; *Conorhinus lectularius* Neiva, 1914; *Triatoma heidemanni* Neiva, 1911, 1914. Blatchley, 1926. Pinto, 1931. Usinger, 1943; *Triatoma occulta* Neiva, 1911, 1914; *Triatoma sanguisuga* Neiva, 1914; *Triatoma lecticularius* Usinger, 1944; *Triatoma lecticularius occulta* Usinger, 1944; *Triatoma lecticularius floridana* Usinger, 1944.

### 3.2. Morphological Characterization of Paratriatoma

Ovoidal shape of the head, scarcely shorter than the pronotum. Eyes rather, small not strongly projected. Anteocular region longer than postocular, transversely impressed behind the strongly elevated clypeus. Ocelli slightly elevated, widely separated. Antennae inserted closer to the eyes than to the apex of the head, as in *Triatoma.* Pronotum somewhat wider than long. Scutellum devoid of a discal depressed area, prolonged into a cylindrical, apical process. Legs rather short, scarcely incrassate, mutic. The entire body and the appendages sparsely covered with long, coarse hairs.

### 3.3. Morphological Characterization of P. lecticularia comb. nov.

Coloration. Overall color piceous, with orange or orange-yellow markings on pronotum, pleura, corium, connexivum, and ventral surface of the abdomen (Figure 1, Figure 2, Figure 3 and Figure 4). Head uniformly dark, neck uniformly light in color (Figure 8). Pronotum dark with 1 + 1 orange bands on lateral margins of the posterior lobe, posterior margin of pronotum is lighter colored in some specimens; in some specimens, dark areas of the posterior lobe of pronotum reduced to one wide median and 1 + 1 sublateral stripes, with orange color extending as 1 + 1 wide lateral and 1 + 1 submedian spots occupying carinae and triangularly widened posteriorly, confluent behind with lateral markings (Figure 3 and Figure 4). Pleura dark. Scutellum dark. Corium with a large central spot of general color, with basal and subapical spots orange-yellow, and the outer border is narrowly bordered with light color. Legs dark, but with coxae in many cases lightened. Venter with orange-yellow markings at the level of light-colored connexival markings, covering intersegmental sutures and extending along lateral borders of urosternites; in some cases, the entire venter tinged with orange, especially on basal half (Figure 1, Figure 3, and Figure 4). Connexival segments dark, posteriorly with orange-yellow spot narrowly extending across intersegmental suture onto the anterior portion of the following segment; extension of light markings variable (Figure 1 and Figure 8B).

**Figure 9:**
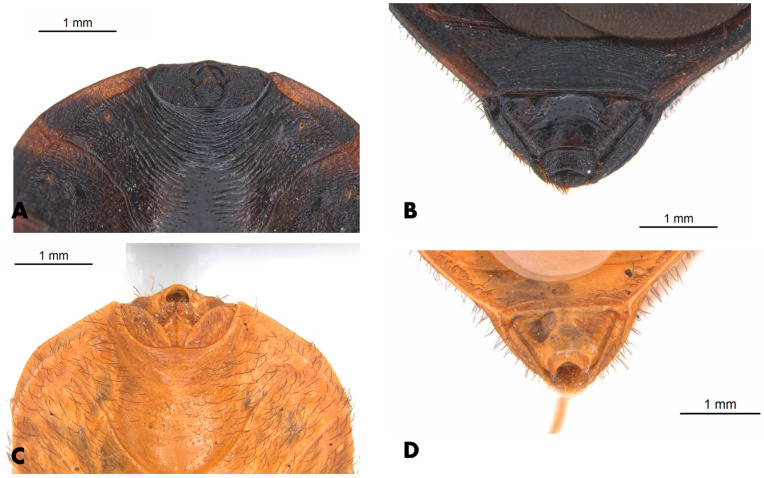
Morphological similarities of the female external genitalia of *P. lecticularia* and *P. hirsuta.* (**A**) Ventral view of external genitalia of *P. lecticularia.* (**B**) Triangular shape of the posterior view of external genitalia of *P. lecticularia.* (**C**) Ventral view of external genitalia of *P. hirsuta.* (**D**) Triangular shape of the posterior view of external genitalia of *P. hirsuta.*

Morphological features. The body surface clothed with distinct decumbent hairs. *Head* (Figure 2 and Figure 8) granulose, ovoidal shape, conspicuously convex; slightly less than twice as long as width across eyes and slightly shorter than pronotum. Anteocular region about three times as long as postocular, postocular with sides convex, converging posteriorly. Eyes in lateral view attaining or slightly surpassing the level of under but not attaining the level of the upper surface of the head. Antenniferous tubercles situated on the posterior third of the anteocular region, comparatively close to the eyes. First antennal segment falling short of apex of clypeus. Rostrum conspicuously hairy, especially on second and third segments; first segment attaining a level of apex of antenniferous tubercle, second attaining level of the neck. The third segment of the rostrum smaller than the first, which is smaller than the second. *Pronotum* (Figure 1), narrow anteriorly, becoming wider posteriorly and extending onto humeri; posterior margin of pronotum possess submedian carinae and collar with anterolateral processes; Anterior lobe without discal or lateral tubercles. Posterior lobe rugose, with conspicuous dark setae on the entire surface; submedian carinae evanescent posteriorly. Humeral angles rounded, slightly elevated. Anterolateral angles short, rounded apically. *Scutellum* (Figure 1) with central portion only very slightly depressed, limited by irregular carinae; apical process shorter than the main body of scutellum, setose, tapering distally, its apex deflected. *Hemelytra* reaching nearly to the apex of the abdomen. *Corium* completely with conspicuous adpressed black setae. *Legs* hairy, rather short. Fore- and mid-femora with 2 + 2 very short denticles subapically. Tibiae of first and second pair in males with small spongy fossulae, absent in the female. *Spiracles* remote from connexival suture (Figure 9). *Venter* conspicuously pilose (Figure 1). *Connexivum* with distinct adpressed setae. In unfed specimens, the plates of the dorsal and ventral connexivum of the urosternite overlap the mesal portion of the ventral plate of the connexivum, with a distinct membrane interpolated between the edge of the connexival plate and the lateral edge of the urosternum (Figure 8). This membrane is not normally visible in museum specimens, in which the ventral plates of the connective appear exceptionally narrow, as they are partially covered by urosternites. Urosternites VIII, IX, and X form the female external genitalia (Figure 9).

A synonymized *T. l. floridana* form is distinguished by its paler coloration and large eyes. A synonymized *T. l. occulta* is an extreme form, smaller in body and eye size.

Vestiture. Pilosity is well developed on the entire body including the corium (Figure 1 and Figure 2).

## 4. Discussion

### 4.1. Transfer of Triatoma lecticularia to Paratriatoma

Stål (1859) described this species as *Conorhinus lecticularius*, and the specimen label states the locality as “India Orientalis” (Figure 1). The insect bears an obviously erroneous locality label because it does not inhabit the East Indies [4]. The type specimen was housed in the Berlin Museum. In 1872, Stål synonymized his *lecticularius* with *variegatus*, a synonym of *rubrofasciata*. Later, Neiva set *lecticularius* in the synonymy of *sanguisuga* [20]. Lent and Wygodzisnky synonymized the subspecies *T. l. occulta* and *T. l. floridana* with *T. lecticularia,* and later appointed a lectotype (Figure 1) [4].

The distribution of *Paratriatoma lecticularia* (Stål, 1859) comb. nov. includes the United States (Arizona, California, Florida, Georgia, Illinois, Kansas, Louisiana, Maryland, Missouri, New Mexico, South Carolina, Tennessee, and Texas) and Mexico (Nuevo Leon), in ecotopes with widely variable climatic conditions, from humid forests to valleys and deserts. The species is found associated with the terrestrial rodent *Neotoma micropus* Baird, and rock squirrel *Spermophilus variagatus* (Erxleben) [21], whereas *Paratriatoma hirsuta* (Figure 6 and Figure 7) is thought to be obligate ectoparasites of the pack rats or wood rats *Neotoma lepida* Thomas, *Neotoma fuscipes macrotis* Thomas, and *Neotoma albigula* Hartley. In human dwellings, *Paratriatoma hirsuta* is found under cracks in the wall, beds, and wooden hollows, and has been found naturally infected by *Trypanosoma cruzi* (Chagas, 1909) [16,21].

Lent and Wygodzinsky determined that the *Triatoma lecticularia* complex included *T. lecticularia*, *T. sanguisuga*, and *T. indictiva* [4] and later Schofield and Galvão [8] added *T. gerstaeckeri, T. recurva, and T. rubida* to the complex. Monteiro et al. [6] proposed that the North American Clade (North of Tehuantepec) of *Triatoma protracta* (Uhler, 1894), *Triatoma barberi* Usinger, 1939, *T. lecticularia*, *Paratriatoma hirsuta* Barber, 1938, and *Dipetalogaster maxima* (Uhler, 1894) comprised a separate species group changing the classification of “complexes” of several species. Nevertheless, as hypothesized by Lent and Wygodzinsky [4], *Paratriatoma* is close to the *Triatoma protracta* complex, so the *T. protracta* complex can be considered one of the sister groups to *Paratriatoma,* despite having the membrane structure of the connexivum apomorphic (Figure 8).

Cytogenetically, the typical autosome number (A) in triatomines is 20. *Paratriatoma lecticularia* differs from other North American species in both chromosome complement and behavior of the sex chromosomes. *Paratriatoma lecticularia* presents the karyotype 2n = 22 (20A + XY)—the same as *Paratriatoma hirsuta*. All the other species in the *T. lecticularia* complex exhibit karyotype 2n = 23 (20A + X1 × 2Y). Considering that *T. rubida* present 2n = 23 and *P. lecticularia* and *P. hirsuta* 2n = 22, it was hypothesized that the ancestor of these triatomines had 22 chromosomes, and during the divergence of *T. rubida* and others, probably an agmatoploidy of the X sex chromosome occurred [14,19].

Justi et al. [3] performed a molecular phylogeny of Triatomini, recovering a clade that includes the genera *Hermanlentia*, *Paratriatoma*, and *Dipetalogaster* + *Linshcosteus* + Northern Hemisphere *Triatoma* (BS = 88, PP = 1); *P. hirsuta* + *P. lecticularia* were considered sister species in the phylogeny. Using Bayesian analysis (with six fossils calibrations), Justi et al. [18] proposed *P. hirsuta* and *P. lecticularia* as sister species (with high support = 1). Kieran et al. [22] analyzed 40 species of Triatomini using Ultraconserved Elements (UCEs) and ribosomal dataset, and based on the best likelihood tree concluded that *Paratriatoma hirsuta* and *P. lecticularia* are sister species and are included in the *protracta* clade.

In addition to the morphological characters mentioned in the results, reconstruction of the ancestral character state of the fossula spongiosa in males puts *P. hirsuta* and *P. lecticularia* in the same clade [22] and, the structure of the plates and membrane of connexivum are characteristic of *Paratriatoma* (Figure 8), being apomorphic with the Triatomini tribe. Combining morphological, molecular, and cytogenetic data, it is clear that *Paratriatoma lecticularia* comb. nov. does not belong to *Triatoma*.

*Paratriatoma lecticularia* occurs from the south-central to the Atlantic coast of the USA and can be collected in houses, kennels, woodrat nests *(Neotoma),* and squirrels. It has been reported as a nuisance species, commonly found in well-built dwellings in central Texas. *Paratriatoma hirsuta* occurs in the western United States, collected from arid regions in woodrat nests and human dwellings, and has been described as “attacking human”, in addition to being of public health importance due to allergic reactions caused by its bite [13,23]. Both species proved to be a competent vector of *T. cruzi* experimentally. Colonization and invasion of dwellings by triatomines is an important factor in vector transmission, as it increases the chance of feeding potentially infected vectors in humans [23,24]. With the marked loss of their habitats, caused by deforestation and unplanned urbanization, major changes in the epidemiology of Chagas disease are expected. However, we know that a strong and sustained surveillance system is the best strategy. In this dynamic world, making predictions is not easy, so with all the knowledge about the biology, morphological, molecular, and cytogenetic taxonomy of triatomine, we can plan public health control in a world with an uncertain future [25].

### 4.2. Dichotomous Key for Species of Paratriatoma

Head, body, and appendages with numerous broad, curved, semi-erect setae; head very strongly convex dorsally; small eyes; antenniferous tubercles inserted near the anterior edge of the eye; anterior femurs without denticles; absent spongy fossulae; legs short and robust, the posterior femurs less than six times longer than broad; first segment of the rostrum less than twice the length of the third; general body coloration light to dark brown *Paratriatoma hirsuta* (Figure 5, Figure 6 and Figure 7).

Head strongly convex dorsally, especially between eyes; antenniferous tubercles elongate, comparatively close to eyes; body clothed with numerous black setae, conspicuous on head; overall color piceous, with orange or orange-yellow markings on pronotum, pleura, corium, connexivum, and ventral surface of abdomen; tibiae of first and second pair in males with small spongy fossulae, absent in female *Paratriatoma lecticularia* (Figure 1, Figure 2, Figure 3 and Figure 4).

## 5. Conclusions

The genus *Paratriatoma* is closely related to the paraphyletic genus *Triatoma*—the most diverse and relevant in the epidemiology of Chagas disease. Initial examination of *Triatoma lecticularia* revealed the hirsuteness covering the entire body—a characteristic and striking feature of members of the genus *Paratriatoma*—and a systematic study revealed several other morphological characters that are in diagnostic alignment with *Paratriatoma*. Molecular phylogeny treatments consistently place the species *Triatoma lecticularia* (Stål, 1859) as sister to the genus *Paratriatoma*. To determine its correct taxonomic assignment, we examined the morphology of several specimens, including types, and cytogenetic data for both taxa. The observations clearly support the formal transferal of *Triatoma lecticularia* (Stål, 1859) into the genus *Paratriatoma* with the resulting new combination: *Paratriatoma lecticularia* (Stål, 1859) comb. nov. (Hemiptera: Reduviidae: Triatominae).

## Figures and Tables

**Table 1:** Information about the specimens examined, including the collection that is deposited.

Scheme 2920	Collection	Additional Information
*Paratriatoma lecticularia* comb. nov.	Humboldt Museum für Naturkunde, Berlin, Germany.	Male, Lectotypus, 2920
*Paratriatoma lecticularia* comb. nov.	José Manuel Ayala-Landa personal collection	San Antonio, TX 78201, USA, Female
*Paratriatoma lecticularia* comb. nov.	Triatomine collection “José Maria Soares Barata”, Universidade Estadual Paulista (UNESP), Brazil.	Male and female, CTA137
*Paratriatoma hirsuta*	U.S. National Entomological Collection, National Museum of Natural History, Smithsonian Institution, Washington DC	Type No. 52747 USNM, UCR_ENT 00007957, Mojave Cal.8.23.35
*Paratriatoma hirsuta*	Coleção de Triatomíneos do Instituto Oswaldo Cruz, Fiocruz, Brazil.	Male, H. Lent det., CTIOC 11976, N° 2729. Female Coleção Rodolfo Carcavallo, n° 1724, CTIOC N° 6229
*Paratriatoma hirsuta*	Coleção Entomológica de Referência, Faculdade de Saúde Pública, Universidade de São Paulo, Brazil	Female Suporte 141, tubo 2 n° E5027 Desert Center, Calif. Riverside Couty 28/11/50. Col. R.E. Ryckman. Female Suporte 141, tubo 1 n° E5026 Desert Center, Calif. Riverside Couty 28/11/50. Col. R.E. Ryckman. Reared 1952
*Paratriatoma hirsuta*	José Manuel Ayala-Landa personal collection	Joshua Tree National Park, USA- California. Female.
